# Probiotics for the Prevention of Antibiotic-Associated Diarrhea in Outpatients—A Systematic Review and Meta-Analysis

**DOI:** 10.3390/antibiotics6040021

**Published:** 2017-10-12

**Authors:** Sara Blaabjerg, Daniel Maribo Artzi, Rune Aabenhus

**Affiliations:** The Research Unit for General Practice and Section of General Practice, University of Copenhagen, 1014 Copenhagen K, Denmark

**Keywords:** primary care, antibiotic-associated diarrhea, probiotics, *Lactobacillus*, *Bifidobacterium*, *Saccharomyces*

## Abstract

A common adverse effect of antibiotic use is diarrhea. Probiotics are living microorganisms, which, upon oral ingestion, may prevent antibiotic-associated diarrhea (AAD) by the normalization of an unbalanced gastrointestinal flora. The objective of this systematic review was to assess the benefits and harms of probiotics used for the prevention of AAD in an outpatient setting. A search of the PubMed database was conducted and yielded a total of 17 RCTs with 3631 participants to be included in the review. A meta-analysis was conducted for the primary outcome: the incidence of AAD. The pooled results found that AAD was present in 8.0% of the probiotic group compared to 17.7% in the control group (RR 0.49, 95% CI 0.36 to 0.66; I^2^ = 58%), and the species-specific results were similar regarding the probiotic strains *L. rhamnosus* GG and *S. boulardii*. However, the overall quality of the included studies was moderate. A meta-analysis of the ten trials reporting adverse events demonstrated no statistically significant differences in the incidence of adverse events between the intervention and control group (RD 0.00, 95% CI −0.02 to 0.02, 2.363 participants). The results suggests that probiotic use may be beneficial in the prevention of AAD among outpatients. Furthermore, the use of probiotics appears safe.

## 1. Introduction

Diarrhea is a common adverse effect of systemic antibiotic treatment. Antibiotic-associated diarrhea (AAD) occurs in 5% to 39% of patients, from the beginning and up to two months after the end of treatment [1]. Any type of antibiotics can cause AAD. In particular, aminopenicillins, cephalosporins, and clindamycin that act on anaerobes are associated with a high risk of AAD [2]. The symptoms range from mild and self-limiting diarrhea to severe diarrhea, the latter particularly in *Clostridium difficile* infections.

The primary care sector is responsible for the bulk of antibiotic consumption in humans [3]. Reports suggest that a major part of this antibiotic use may, in fact, be inappropriate, and efforts to reduce and target antibiotics are rightly promoted. However, when antibiotic therapy is deemed necessary, it is useful to have an easily available, cost effective, and safe method to prevent side effects associated with the issued antibiotic.

Probiotics are defined as “live microorganisms which when administered in adequate amounts confer a health benefit on the host” [4]. The rationale behind the administration of probiotics in gastrointestinal disorders is based on the hypothesis that they may assist a normalization of an unbalanced gastrointestinal flora. There are many proposed mechanisms by which probiotics enhance intestinal health, including the stimulation of immunity, competition for nutrients, the inhibition of the epithelial and mucosal adherence of pathogens, the inhibition of epithelial invasion, and the production of antimicrobial substances [5].

Numerous probiotic species have been tested, most commonly the *Lactobacillus* genus, *Bifidobacterium* genus, and *Saccharomyces* genus. Previous reviews suggest that probiotics are useful in the prevention of AAD, especially in a pediatric population (RR 0.46; 95% CI 0.35 to 0.61) with a NNT of 10 [6]. However, these reviews have mainly focused on the prevention of AAD in inpatients from secondary care settings, which was likely influenced by the intensity of antibiotic treatment (intravenous vs. oral), the type of infection, and the microbial pathogens, in turn making the translation of the results into the primary care sector less straightforward.

The objective of this systematic review and meta-analysis was thus to assess the benefits and harms of probiotics used for the prevention of antibiotic-associated diarrhea in outpatients of all ages.

## 2. Results

### 2.1. Description of Studies

#### 2.1.1. Results of the Search

A total of 637 studies were identified through MEDLINE/PubMed. An independent review of these titles and abstracts identified 53 potentially relevant studies for full-text reviews. Of these studies, 17 met the inclusion criteria. The details of the study flow, including reasons for exclusion, are documented in the study flow diagram in Supplementary Materials File S1.

#### 2.1.2. Design

All included studies were prospective, randomized, controlled trials with placebo, active, or no treatment control arms. Additional information about each study can be found in the Characteristics of Included Studies table in Supplementary Materials File S2.

#### 2.1.3. Patient Population

The 17 studies included a total of 3631 patients. The patient population was restricted to outpatients taking oral antibiotics, as trials randomizing hospitalized patients were excluded. The recruitment and evaluation of patients took place in private practices, pharmacies, or hospitals (ambulatory settings, outpatient clinics, etc.).

All studies included both males and females.

#### 2.1.4. Interventions

Probiotics:

The trials tested the prevention of AAD with *Lactobacilli* spp., *Lactococcus* spp., *Bacillus* spp., *Bifidobacterium* spp., *Saccharomyces* spp., *Leuconostoc cremoris*, *Clostridium* spp., or *Streptococcus* spp. Eight studies (*N* = 1638; Tankanow 1990, Park 2007, Conway 2007, Kim 2008, Merenstein 2009, De Vrese 2011, Chatterjee 2013, Fox 2014) used a combination of two or more probiotic strains as intervention. Additional information about the type of probiotic(s) that were used, including the dosages and treatment durations, can be found in Table 1.

Antibiotics:

The patients were treated with oral antibiotics for various clinical indications, but the most common reason was *H. pylori* eradication with a combination of clarithromycin and amoxicillin (seven studies: *N* = 1450; Duman 2005, Park 2007, Cindoruk 2007, Imase 2008, Kim 2008, De Vrese 2011, Zojaji 2013). One study (Ojetti 2012) used a combination of levofloxacin and amoxicillin for *H. pylori* eradication therapy. Two studies (Tankanow 1990, Erdeve 2004) reported the use of single beta-lactam antibiotics, while others included several antibiotics or were otherwise unspecified.

Aside from *H. pylori* infection, the most common indications for treatment with antibiotics were upper and lower respiratory tract infections, otitis media, and throat infections.

#### 2.1.5. Comparison

Most RCTs randomized a moderate number of participants (median, 174.0; mean [SD], 205.3 [130.2]) to either probiotics vs. placebo (nine studies: *N* = 1557; Tankanow 1990, Arvola 1999, Vanderhoof 1999, Cindoruk 2007, Merenstein 2009, De Vrese 2011, Chatterjee 2013, Fox 2014, Olek 2017) or probiotics vs. no treatment (eight studies: *N* = 2074; Erdeve 2004, Duman 2005, Park 2007, Conway 2007, Kim 2008, Imase 2008, Ojetti 2012, Zojaji 2013).

#### 2.1.6. Outcomes

All of the included studies provided data on the main outcome: the incidence of AAD. The outcomes were patient-reported. The definitions of diarrhea varied in each study in regard to the number of bowel movements per day and the consistency of stools (“semi-solid”, “watery”, “liquid”, “abnormally loose”, etc.). Seven studies (*N* = 1724; Arvola 1999, Duman 2005, Conway 2007, De Vrese 2011, Chatterjee 2013, Fox 2014, Olek 2017) applied the WHO definition of diarrhea (“the passage of three or more loose or liquid stools per day”) [24]. Three studies (*N* = 561; Cindoruk 2007, Kim 2008, Ojetti 2012) categorized diarrhea into groups (“none”, “mild”, “moderate”, and “severe”), but did not include the frequency or consistency of bowel movements. Four studies (*N* = 656; Park 2007, Imase 2008, Merenstein 2009, Zojaji 2013) did not provide any definition of diarrhea. The individual studies’ definitions of diarrhea can be seen in Table 2.

Ten studies (*N* = 2363; Tankanow 1990, Arvola 1999, Vanderhoof 1999, Duman 2005, Conway 2007, Kim 2008, Merenstein 2009, Chatterjee 2013, Fox 2014, Olek 2017) reported the incidence of adverse events (i.e., the number of participants with at least one adverse event of any type). The definitions of adverse events varied widely. Four studies (*N* = 762; Vanderhoof 1999, Arvola 1999, De Vrese 2011, Chatterjee 2013) reported the mean duration of diarrhea, but the data was not sufficient to make a quantitative analysis of this outcome. Instead these are summarized qualitatively and/or by descriptive statistics.

### 2.2. Risk of Bias in Included Studies

**Figure 1:**
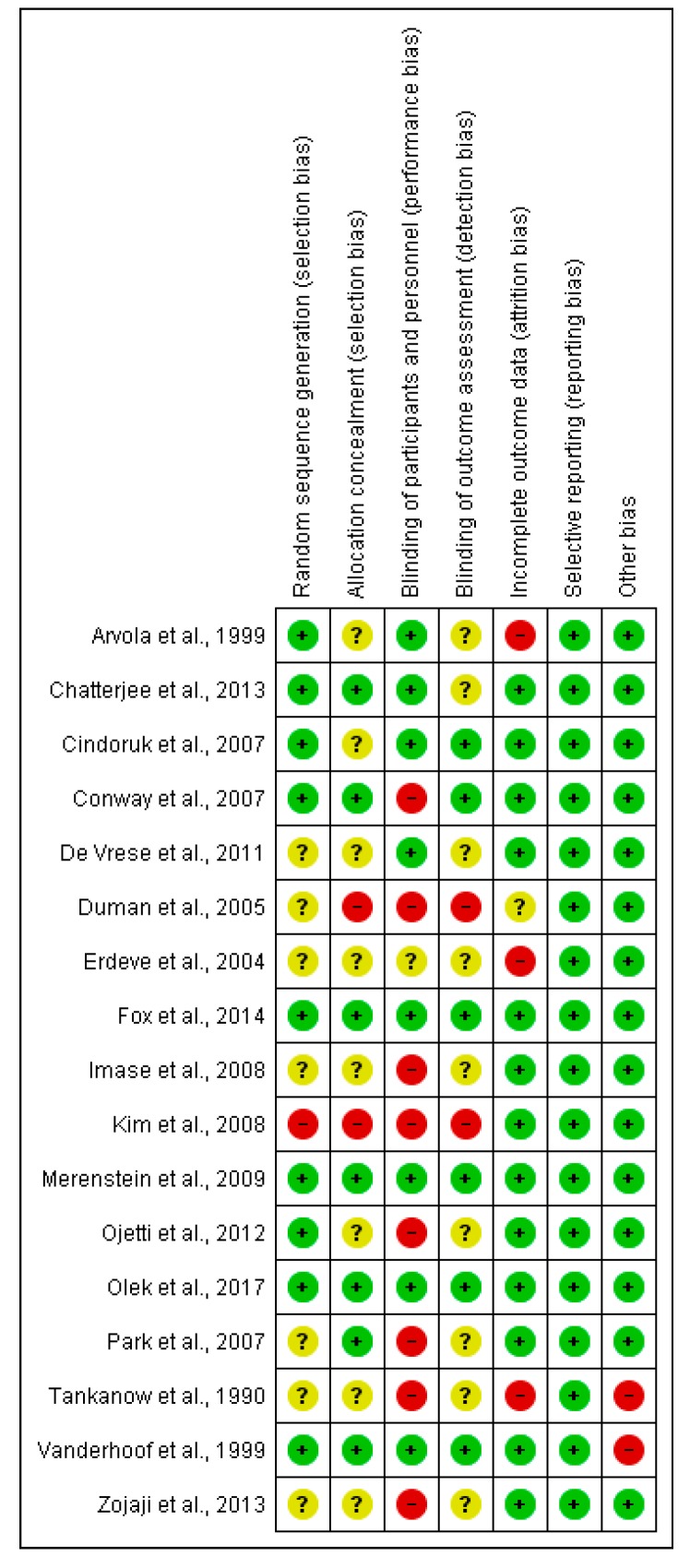
Risk of bias summary.

The risk of bias is categorized into three categories: high risk of bias, low risk of bias, and unclear. The individual studies’ results of the risk of bias assessment are shown in Figure 1.

The quality of reporting was low; 11 trials lacked adequate information to assess one or more of the parameters, thus making the risk of bias “unclear”. This was the case particularly regarding allocation concealment and blinding methods. For the blinding of participants, nearly half of the studies were evaluated as having a “high risk of bias” because the participants in the control group did not receive any kind of placebo matching the probiotic(s) given to the intervention group.

Loss to follow-up was substantial (i.e., >20%) in three trials (Tankanow 1990, Arvola 1999, Erdeve 2004). 11 studies (Tankanow 1990, Arvola 1999, Vanderhoof 1999, Erdeve 2004, Duman 2005, Cindoruk 2007, Imase 2008, De Vrese 2011, Zojaji 2013, Fox 2014) did not perform an intention-to-treat analysis.

**Figure 2:**
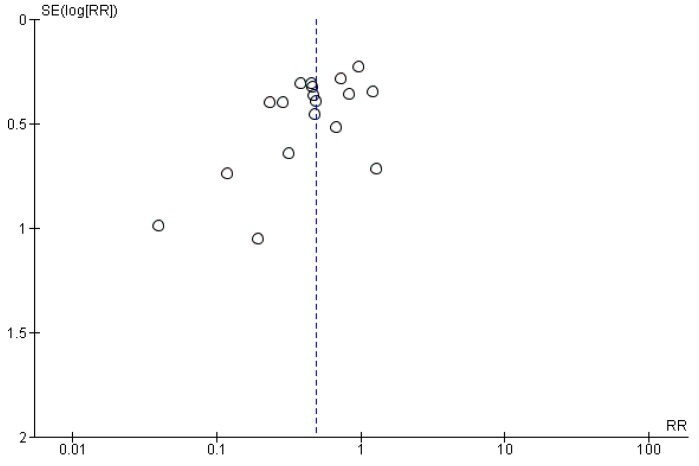
Funnel plot.

Visual inspection of the funnel plot (Figure 2) for the primary outcome identified minor asymmetries for the smaller studies, but the relationship between the risk ratio and standard error did not appear substantially skewed, in turn suggesting that a possible publication bias is not likely to markedly affect the results.

### 2.3. Effects of Interventions

#### 2.3.1. Main Outcome: Incidence of Antibiotic-Associated Diarrhea

All of the 17 included studies reported the incidence of diarrhea and the number of patients randomized to each group. The incidence of AAD in the probiotic group was 8.0%, compared to 17.7% in the control group. The overall pooled results showed that the use of probiotics produced a statistically significant reduction in the incidence of AAD: RR 0.49; 95% CI 0.36 to 0.66. The forest plot can be seen in Supplementary Materials File S3.

Statistically significant heterogeneity was detected (*p* = 0.001), and this was moderate (I^2^ = 58%). A GRADE analysis (Supplementary Materials File S4) indicated that the overall quality of evidence for this outcome was moderate due to moderate heterogeneity and a high risk of bias in trials.

**Figure 3:**
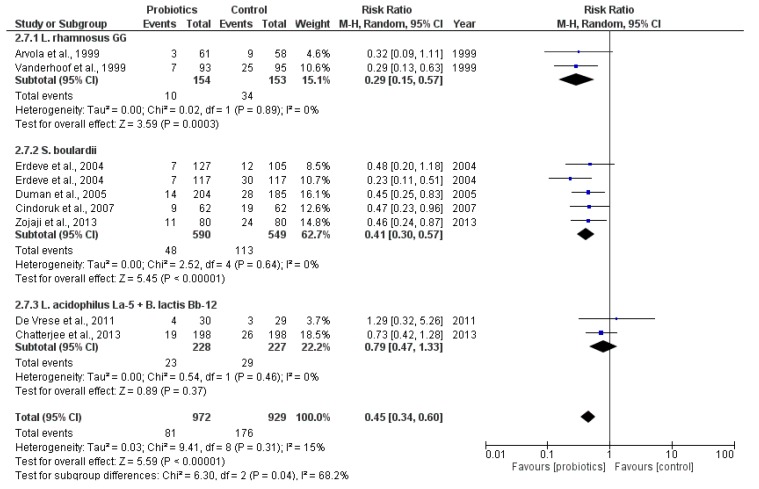
Efficacy results of probiotic use: eight RCTs by three probiotic subgroups (outcome: incidence of antibiotic-associated diarrhea (AAD)).

In addition to the pooled analysis of any probiotic, a strain-specific meta-analysis (Figure 3) was conducted on eight of the included studies with three subgroups of the following probiotic strain(s): two studies using *L. rhamnosus GG* (*N* = 307; Arvola 1999, Vanderhoof 1999), four studies using *S. boulardii* (*N* = 1139; Erdeve 2004, Duman 2005, Cindoruk 2007, Zojaji 2013), and two studies using a combination of *L. acidophilus La-5* and *B. lactis Bb-12* (*N* = 455; De Vrese 2011, Chatterjee 2013).

The results were similar to the overall pooled analysis and showed a beneficial effect of probiotics in the prevention of AAD. This effect was statistically significant in two of the three subgroups.

The subgroup analyses on *L. rhamnosus GG* and *S. boulardii* showed a statistically significant lower risk of AAD, while this was not the case regarding the combination probiotic supplement of *L. acidophilus La-5* and *B. lactis Bb-12* (RR 0.79; 95% CI 0.47 to 1.33). The high level of heterogeneity in the overall pooled results could no longer be detected in these three subgroups (I^2^ = 0%). A GRADE analysis (Supplementary Materials File S4) indicated that the overall quality of evidence for this outcome was high.

#### 2.3.2. Secondary Outcome: Incidence of Antibiotic-Associated Diarrhea Using the Criteria Defined by WHO

**Figure 4:**
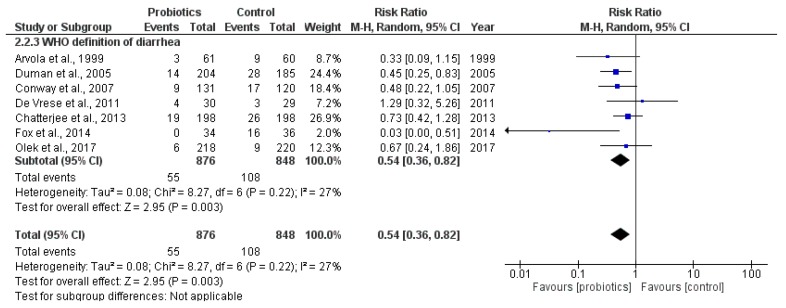
Efficacy results of probiotic use by study (secondary outcome: incidence of AAD using the criteria defined by WHO).

Seven studies (*N* = 1724; Arvola 1999, Duman 2005, Conway 2007, De Vrese 2011, Chatterjee 2013, Fox 2014, Olek 2017) applied the WHO definition of diarrhea, and the pooled results showed that the use of probiotics produced a statistically significant reduction in the incidence of AAD: RR 0.54; 95% CI 0.36 to 0.82 (Figure 4).

No statistically significant heterogeneity was detected (*p* = 0.22; I^2^ = 27%). A GRADE analysis (Supplementary Materials File S4) indicated that the overall quality of evidence for this outcome was high.

#### 2.3.3. Secondary Outcome: Mean Duration of Diarrhea

Four studies (*N* = 762; Vanderhoof 1999, Arvola 1999, De Vrese 2011, Chatterjee 2013) reported the mean duration of diarrhea (MDD). The results can be seen in Table 3. The standard deviations (SD) for these trials were not reported so a quantitative analysis for this outcome was not possible. One study (Arvola 1999) showed a similar duration of diarrhea in both groups, while the other three all showed a positive effect on the MDD in the intervention group. Combining these four studies, the average MDD in the intervention group was 2.93 days, while the average was 4.65 days in the control group.

#### 2.3.4. Secondary Outcome: Incidence of Adverse Events

None of the 17 included studies specifically defined adverse events prior to enrolment of participants. Ten trials (*N* = 2363; Tankanow 1990, Arvola 1999, Vanderhoof 1999, Duman 2005, Conway 2007, Kim 2008, Merenstein 2009, Chatterjee 2013, Fox 2014, Olek 2017) reported the number of participants with adverse events in each group. Adverse events consisted of a variety of different symptoms such as metallic taste, nausea, loss of appetite, epigastric discomfort, headache, flu-like symptoms, rash, etc. There were no serious adverse effects leading to major disabilities, hospitalization, or death. Three trials (Kim 2008, Fox 2004, Olek 2017) found a statistically significant difference in adverse events between groups; one favors placebo and two favor probiotics. Kim et al. found a difference in the presence of metallic taste between the intervention group (16.7%) and the control group (7.3%). Fox et al., as well as *Olek* et al., reported more adverse events in their placebo-controlled patient group, and these were abdominal pain, loss of appetite, nausea, pyrexia, headache, and rash.

**Figure 5:**
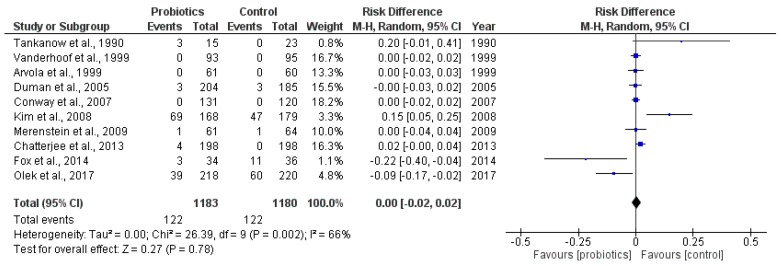
Adverse events.

A meta-analysis of the ten trials reporting on any adverse events (Figure 5) demonstrated no statistically significant differences in the incidence of adverse events between the intervention and control groups (RD 0.00, 95% CI −0.02 to 0.02, 2363 participants).

A GRADE analysis indicated that the overall quality of evidence for this outcome was low due to heterogeneity and indirectness (Supplementary Materials File S4).

#### 2.3.5. Dose-Response Analysis

The analysis regarding dose-response relationships included ten studies of different probiotic species. The pooled effect size for doses larger than 5 × 10^9^ CFU/day was RR 0.18 (95% CI 0.08 to 0.42; I^2^ = 41%), and, for doses less than 5 × 10^9^ CFU/day, it was RR 0.61 (95% CI 0.42 to 0.90; I^2^ = 40%). The difference in response rates between high-dose and low-dose probiotics were statistically significant (*p* < 0.002) by Fisher's exact test). The forest plot is provided in Supplementary Materials File S5.

### 2.4. Subgroup Analyses

Prespecified subgroup analyses for the main outcome were conducted on (1) age groups; (2) trials with *H. pylori* eradication; (3) low risk of bias; and (4) intention-to-treat analyses. Forest plots from each subgroup analysis can be seen in (Supplementary Materials Files S6–S9).

#### 2.4.1. Age Groups

Two subgroups were based on the age of the participants, including children (<15 years of age) and adults (>15 years of age). Seven trials (*N* = 1446; Tankanow 1990, Arvola 1999, Vanderhoof 1999, Erdeve 2004, Merenstein 2009, Fox 2014, Olek 2017) targeted children specifically, and their results showed a statistically significant lower risk of AAD but with considerable heterogeneity (RR 0.42; 95% CI 0.23 to 0.77; I^2^ = 76%).

Nine trials (*N* = 1936; Duman 2005, Park 2007, Cindoruk 2007, Imase 2008, De Vrese 2011, Ojetti 2012, Zojaji 2013, Chatterjee 2013) targeting adults also showed a statistically significant reduction in the risk of AAD and moderate heterogeneity (RR 0.53; 95% CI 0.37 to 0.76; I^2^ = 47%).

One trial (Conway 2007) included both children and adults and therefore was not included in this subgroup analysis.

#### 2.4.2. Trials with *H. pylori* Eradication Therapy

In the seven trials using a combination of clarithromycin and amoxicillin for *H. pylori* eradication therapy (*N* = 1450; Duman 2005, Park 2007, Cindoruk 2007, Kim 2008, Imase 2008, De Vrese 2011, Zojaji 2013), adjunct probiotic use was also associated with a lower risk of AAD (RR 0.52; 95% CI 0.32 to 0.85; I^2^ = 53%).

#### 2.4.3. Low Risk of Bias

The trial quality was generally low, and only three studies (*N* = 633; Merenstein 2009, Fox 2014, Olek 2017) were evaluated as having a low risk of bias. Combining these three low risk studies in a meta-analysis showed a non-significant lower risk of AAD and considerable heterogeneity (RR 0.36; 95% CI 0.08 to 1.64; I^2^ = 82%).

#### 2.4.4. Intention-To-Treat Analyses

A meta-analysis combining the six studies using intention-to-treat analyses (*N* = 1561; Park 2007, Conway 2007, Kim 2008, Merenstein 2009, Ojetti 2012, Chatterjee 2013) showed similar results to the overall pooled analysis, with a significantly lower risk of AAD and moderate heterogeneity (RR 0.58; 95% CI 0.36 to 0.94; I^2^ = 60%).

## 3. Discussion

The results of this review point towards a protective effect of the use of probiotics as adjunct therapy to prevent antibiotic-associated diarrhea in outpatients of all ages. Data from 17 studies with a total of 3631 patients found that the use of a probiotic may reduce the risk of AAD by 51% (RR 0.49; 95% CI 0.36 to 0.66; I^2^ = 58%), with no apparent increase in the risk of side effects (RD 0.00, 95% CI −0.02 to 0.02, 2.363 participants). The number needed to treat (NNT) to prevent one case of diarrhea was 11 (95% CI 6 to 13). The quality of evidence for the main outcome was categorized as moderate due to a moderate degree of heterogeneity and a high risk of bias in some trials.

A strain-specific subgroup analysis combining data from eight of the included trials showed a similar protective effect of probiotics in the prevention of AAD when compared to the overall pooled analysis. The most effective probiotic strain was *L. rhamnosus GG* (RR 0.29; 95% CI 0.15 to 0.57; 307 participants), followed by *S. boulardii* (RR 0.41; 95% CI 0.30 to 0.57; 1.139 participants). Furthermore, with this subgroup analysis, the heterogeneity from the pooled analysis (I^2^ = 58%) disappeared in each of the three subgroups (I^2^ = 0%).

Data from the seven studies applying the definition of diarrhea defined by WHO showed a similar protective effect of probiotic use to prevent AAD (RR 0.54; 95% CI 0.36 to 0.82) but with no statistically significant heterogeneity (I^2^ = 27%; *p* = 0.22). This explains some of the statistical heterogeneity, and it also demonstrates the importance of having clear and consistent definitions of outcomes in clinical trials. The quality of evidence for this outcome was categorized as high.

We also provide preliminary evidence of a possible dose-response relationship, as results indicate that higher doses were associated with fewer ADD events (higher than 5 × 10^9^ CFU 3.6% vs. less than 5 × 10^9^ CFU 8.9%; *p* < 0.002). However, this result should be interpreted with caution as the analysis was on any probiotic species and not on specific strains. A review investigating different treatment regimens of probiotics in human studies concluded that a dose-response relationship exists within the commonly studied range of 10^8^ to 10^11^ CFU, meaning that, within this range, a higher dose will lead to a better response [25]. However, the previously-mentioned Cochrane review on the prevention of pediatric AAD did not find any statistically significant difference in the use of high versus low dose probiotics (over or under 5 × 10^9^ CFU/day) [6].

We did not find evidence to suggest an increase in effect when more than one probiotic strain was used to prevent AAD.

Our result was fairly consistent across a number of subgroup analyses in which RRs ranged from 0.36 to 0.58. All but two subgroup analyses yielded a statistically significant result. Of note, the analysis of studies with a low risk of bias did not produce a statistically significant result. This is concerning, and although in part may be ascribed to a low number of trials (three), this finding calls for caution in its interpretation. Nevertheless, our results are in line with a previous Cochrane review [6] on the prevention of pediatric AAD (RR 0.46, 95% CI 0.35 to 0.61, I^2^ = 55%, 3898 participants), as well as a review, including hospitalized patients [26], on the prevention and treatment of AAD regardless of age (RR 0.58, 95% CI 0.50 to 0.68, I^2^ = 54%, 11,811 participants).

Subgroup analyses did not further explain the substantial amount of heterogeneity across studies as heterogeneity remained evident throughout all these analyses. Combining data into a meta-analysis by probiotic species and strain level from all included studies would have been preferred, but this was not possible due to varying species, strains, and combinations of strains used in the included studies.

In most of the included studies, the types of infections/diagnoses of the subjects in the included studies were not specified. This was due to inadequate reporting of the trials. Likewise, the antibiotics used were rarely specified, but, by excluding inpatients from the analysis, some similarity regarding the diagnoses of subjects can be expected. The five most important causes of antibacterial prescribing in primary care are upper respiratory tract infection, lower respiratory tract infection, sore throat, urinary tract infection, and otitis media [27]. Outpatients being prescribed antibiotics are likely to experience less severe and relatively common types of infections than inpatients because the latter requires hospitalization. Also, outpatients were not exposed to intravenous antibiotics. The decision to include only outpatients was made in order to lower the degree of heterogeneity and to have a patient group that more closely represents primary care patients.

Probiotics can be found in the form of yoghurt, tablets, and capsules, e.g., in dietary supplements and as non-prescription drugs from pharmacies. This makes the use of probiotics an easily available and relatively simple method of AAD prophylaxis. Furthermore, the ingestion of probiotics seems safe, and our meta-analysis found no increased risk of adverse events, including serious adverse events. This result is in line with a previous review on the safety of probiotics [28]. The majority of adverse events that occurred such as abdominal pain, loss of appetite, nausea, headache and flu-like symptoms were most likely due to antibiotic side effects or were symptoms from the underlying infection.

## 4. Materials and Methods

### 4.1. Criteria for Selecting Studies for This Review

#### 4.1.1. Types of Studies

All randomized controlled trials in which probiotics were given to prevent antibiotic-associated diarrhea and in which the use of probiotics was compared to either a placebo or an active alternative prophylaxis or in which no treatment were considered for inclusion. Trials were also included if probiotics were given together with antibiotics in *H. pylori* eradication, if the incidence of AAD was reported. Trials testing probiotics for the treatment of diarrhea were not included.

#### 4.1.2. Types of Participants

Studies with outpatients of all ages being administered antibiotic therapy for any indication were considered for inclusion. An outpatient can be defined as “a person who goes to a health-care facility for a consultation, and who leaves the facility within three hours of the start of consultation. An outpatient is not formally admitted to the facility” [29]. Trials with *H. pylori* eradication therapy for otherwise healthy adults were also included, and the subjects were assumed to be outpatients (if not directly stated as inpatients) due to the nature of the trials because this kind of treatment is normally done in an ambulatory setting.

Trials with inpatients were not included in this review because this patient group a priori was different with regard to the severity of their illness, the presence of comorbidity, and equally more comprehensive treatment (e.g., administration of broad-spectrum antibiotics given intravenously to an inpatient vs. narrow-spectrum antibiotics taken orally by an outpatient).

#### 4.1.3. Types of Interventions

Intervention:

The administration of an identified probiotic agent of any specified strain or dose, regardless of the administration form (e.g., yoghurt, capsules, tablets, etc.).

Control:

Administration of placebo or an active comparator or no treatment.

#### 4.1.4. Types of Outcome Measures

Primary outcome:

- Incidence of antibiotic-associated diarrhea (AAD)

This analysis used the original study’s definition of diarrhea.

An overall pooled meta-analysis, as well as a strain-specific subgroup analysis, was conducted.

Secondary outcomes:

- Incidence of AAD using the criteria defined by WHO:

This analysis used the definition of diarrhea authored by WHO. Diarrhea is defined as “the passage of three or more loose or liquid stools per day (or more frequent passage than is normal for the individual)” [24]. Antibiotic-associated diarrhea was considered in cases of a subject having diarrhea in relation to their treatment with antibiotics. A specific time factor in this regard was not considered.

- Mean duration of diarrhea (MDD) in days
- Number and types of adverse events

### 4.2. Search Methods for Identification of Studies

On the 20th of July 2017 a search was conducted of the MEDLINE/PubMed database to identify relevant RCTs. Combinations of the keywords “probiotics”, “prevention”, “antibiotics”, and “diarrhea” were used.

The exact search terms for PubMed can be seen in Supplementary Materials File S10.

### 4.3. Data Collection and Analysis

#### 4.3.1. Study Selection

Two independent investigators screened all the titles and abstracts from the search results and retrieved the relevant articles. The articles were then assessed for inclusion according to the selection criteria defined previously.

#### 4.3.2. Data Extraction and Management

Data extraction and management was conducted by two independent investigators. The following data were extracted from each study: author, year of publication, patient characteristics (age group and mean age), country, study setting, diagnosis, antibiotic(s) administered, probiotic(s) used, comparator, outcome measures (incidence of diarrhea, mean duration of diarrhea, number and type of adverse events), the study’s definition of diarrhea, the number of patients allocated to each group, the presence/absence of intention-to-treat analysis, and the number of participants lost to follow-up or withdrawn from the study (including the reasons for this).

#### 4.3.3. Quality Assessments

Methodological quality assessment using the Cochrane Collaboration’s tool for assessing risk of bias [30] was also done by two independent investigators. Each of the included studies were evaluated for sequence generation, allocation concealment, blinding of participants and personnel, blinding of outcome assessment, incomplete outcome data, selective outcome reporting, and other sources of bias. The risk of bias was visualized in a risk of bias summary and a risk of bias graph.

The overall quality of the evidence supporting the main outcome (incidence of AAD) and the secondary outcomes (incidence of AAD using the criteria defined by WHO; adverse events) was evaluated using “the Grading of Recommendations, Assessment, Development and Evaluations” (GRADE) criteria [31]. RCTs are by default regarded as high quality evidence but may be downgraded on the basis of five categories of limitations: (1) risk of bias; (2) inconsistency; (3) indirectness; (4) imprecision; and (5) publication bias. The quality of evidence can be categorized as either high (we are very confident that the true effect lies close to the estimated effect); moderate (we are moderately confident in the effect estimate; the true effect is likely to be close to the estimated effect, but there is a possibility that new evidence will affect the estimated effect size); low (our confidence in the effect estimate is limited; new evidence may be substantially different from the estimated effect); very low (we have very little confidence in the effect estimate; the true effect is likely to be substantially different from the estimated effect). The data were entered into GRADEpro GDT [32] for analysis of the quality of evidence.

#### 4.3.4. Statistical Analysis

Dichotomous data (antibiotic-associated diarrhea vs. no diarrhea and adverse events vs. no adverse events) were combined in a random-effects meta-analysis using a pooled risk ratio (RR) or risk difference (RD) along with the corresponding 95% confidence interval (95% CI). All models were weighted based on study size. The number needed to treat (NNT) was calculated for the main outcome. The data were entered into Review Manager 5.3 [33] for statistical analysis.

A high degree of heterogeneity was expected because of the broad inclusion criteria (e.g., differences in types of participants, interventions, and definitions of AAD), and this was investigated using the I^2^ statistic, wherein a value of >50% may represent substantial heterogeneity [34]. To explore possible explanations for heterogeneity, prespecified subgroup analyses were conducted on (1) the WHO definition of diarrhea; (2) age groups; (3) studies with *H. pylori* eradication; (4) probiotic genus; (5) low risk of bias; and (6) intention-to-treat analyses.

To evaluate the potential for publication bias, a funnel plot was applied for the efficacy outcome (incidence of AAD).

## 5. Conclusions

Using probiotics for the prevention of antibiotic-associated diarrhea reduces the risk of AAD by 51% (RR 0.49; 95% CI 0.36 to 0.67) with a moderate quality of evidence according to GRADE. This result was confirmed in analyses of specific strains, namely *Lactobacillus rhamnosus GG* and *Saccharomyces boulardii*. Furthermore, we found preliminary evidence to suggest a dose-response relationship.

The use of probiotics appears safe. However, our study still suggests that caution be applied prior to widespread introduction of probiotic treatment for AAD as only 18% of the included studies had a low risk of bias, and these studies did not find a statistical significant reduction in the prevention of AAD.

Limitations to the findings include the paucity of data on probiotic strain level, and future studies on probiotics to prevent AAD should focus on identifying the most effective agent(s) preferable in head-to-head comparisons and follow a stringent approach to definitions of outcomes, as well as clinical scenarios, prior to the widespread recommendation of probiotics as adjunct therapy to antibiotics. Also, more data are needed to determine the safety of probiotics, and trials should define potential adverse events in advance.

## Figures and Tables

**Table 1:** Probiotic(s) used, dosages, and treatment durations.

RCT	Probiotic(s) Used (Genus and Strain)	Dosage	Duration of Treatment
Tankanow et al., 1990 [7]	*Lactobacillus acidophilus* *Lactobacillus bulgaricus*	5.1 × 10^8^ CFU, four times daily	10 days
Vanderhoof et al., 1999 [8]	*Lactobacillus rhamnosus GG*	Children < 12 kg: 1 × 10^10^ CFU, once daily Children > 12 kg: 2 × 10^10^, once daily	10 days
Arvola et al., 1999 [9]	*Lactobacillus rhamnosus GG*	2 × 10^10^ CFU, twice daily	Seven to 10 days
Erdeve et al., 2004 [10]	*Saccharomyces boulardii*	Not mentioned	Not mentioned
Duman et al., 2005 [11]	*Saccharomyces boulardii*	500 mg, twice daily	14 days
Park et al., 2007 [12]	*Bacillus subtilis* *Streptococcus faecium*	two capsules three times a day: 2.5 × 10^9^ CFU (*Bacillus subtilis*) 22.5 × 10^9^ CFU (*Streptococcus faecium*)	Eight weeks
Cindoruk et al., 2007 [13]	*Saccharomyces boulardii*	500 mg, twice daily	14 days
Conway et al., 2007 [14]	*Lactobacillus acidophilus*	10^9^ CFU, once daily	12 days
	*Streptococcus thermophilus*		
	*Bifidobacterium animalis lactis*		
Imase et al., 2008 [15]	*Clostridium butyricum*	1 × 10^7^ CFU per tablet Group B: two tablets, three times daily Group C: 4 tablets, three times daily	Seven days
Kim et al., 2008 [16]	*Lactobacillus acidophilus* *Lactobacillus casei* *Bifidobacterium longum* *Streptococcus thermophilus*	One bottle (150 mL) per day: >1 × 10^5^ CFU/mL (*L. acidophilus*) >1 × 10^5^ CFU/mL (*L. casei*) >1 × 10^6^ CFU/mL (*B. longum*) >1 × 10^8^ CFU/mL (*S. themophilus*)	At least three weeks
Merenstein et al., 2009 [17]	*Lactococcus lactis* *Lactococcus plantarum* *Lactococcus rhamnosus* *Lactococcus casei* *Lactococcus lactis subspecies diacetylactis* *Leuconostoc cremoris* *Bifidobacterium longum* *Bifidobacterium breve* *Lactobacillus acidophilus* *Saccharomyces florentinus*	One bottle (150 mL) per day, amount of CFU not mentioned	10 days
De Vrese et al., 2011 [18]	*Lactobacillus acidophilus LA-5* *Bifidobacterium lactis BB-12*	>1 × 10^6^ CFU/g, 125 g, twice daily	Five weeks
Ojetti et al., 2013 [19]	*Lactobacillus reuteri*	1 × 10^8^ CFU, three times daily	14 days
Chatterjee et al., 2013 [20]	*Lactobacillus acidophilus La-5,*	4 × 10^9^ CFU	14 days
	*Bifidobacterium Bb-12*		
Zojaji et al., 2013 [21]	*Saccharomyces boulardii*	250 mg twice daily, amount of CFU not mentioned	14 days
Fox et al., 2014 [22]	*Lactobacillus rhamnosus, G.G.;* *Lactobacillus acidophilus LA-5*, *Bifidobacterium Bb-12*	5.2 × 10^9^ CFU (*L. rhamnosus*) 5.9 × 10^9^ CFU (*B. Bb-12*) 8.3 × 10^9^ CFU (*L. acidophilus LA-5*)	Number of days not mentioned (“From the start to the end of their antibiotic treatment”)
Olek et al., 2017 [23]	*Lactobacillus plantarum* DSM9843 (LP299V)	1 × 10^10^ CFU/capsule	Five to 10 days during antibiotic treatment and one week after (± two days)

**Table 2:** The individual studies’ definitions of diarrhea.

RCT	Definition of Diarrhea
Tankanow et al., 1990	One or more abnormally loose bowel movements/day throughout the study period of one to 10 days (parental reports)
Vanderhoof et al., 1999	The presence of at least two liquid stools/day during at least two observation periods during the course of the study
Arvola et al., 1999	At least three watery or loose stools/day for a minimum of two consecutive days
Erdeve et al., 2004	Three or more watery stools/day during antibiotic treatment
Duman et al., 2005	A change in bowel habits with at least three semi-solid or watery bowel movements/day for at least two consecutive days
Park et al., 2007	Not specified (self-report)
Cindoruk et al., 2007	Not specified (modified *De Boer* questionnaire categorizing diarrhea into “none”, “mild”, “moderate” and “severe”)
Conway et al., 2007	Three or more loose stools/day over at least two consecutive days during the 12-day follow-up period
Imase et al., 2008	“Loose or mostly loose stools”, not specified further
Kim et al., 2008	Not specified other than categorized in groups (“none”, “mild”, “moderate”, “severe”)
Merenstein et al., 2009	Not specified (parental reports)
De Vrese et al., 2011	Three or more watery stools for at least one day (where at least one episode lay within the eradication week)
Ojetti et al., 2013	Not specified other than categorized in groups (“none”, “mild”, “moderate”, “severe”)
Chatterjee et al., 2013	Passage of at least three or more watery or loose stools/day for at least two consecutive days
Zojaji et al., 2013	Not specified (self-report)
Fox et al., 2014	Categories:
	“A” (stool consistency ≥ 5, ≥2 stools/day for ≥2 days)
	“B” (stool consistency ≥ 5, ≥3 stools/day for ≥2 days)
	“C” (stool consistency ≥ 6, ≥2 stools/day for ≥2 days)
	“D” (stool consistency ≥ 6, ≥3 stools/day for ≥2 days)
Olek et al., 2017	≥3 loose/watery stools/24 h starting after the initiation of antibiotic treatment

**Table 3:** Mean duration of diarrhea (MDD).

	MDD (Days)	Range	Probiotic Group (N)	MDD (Days)	Range	Control Group (N)
Vanderhoof et al., 1999	4.70	N/A	93	5.88	N/A	95
Arvola et al., 1999	4.00	2–8	61	4.00	2–8	58
De Vrese et al., 2011	1.00	N/A	30	4.70	N/A	29
Chatterjee et al., 2013	2.00	1–3	198	4.00	3–5.5	198

## Supplementary Materials

The following are available online at www.mdpi.com/2079-6382/6/4/21/s1, File S1: Study flow diagram, File S2: Characteristics of included studies (RevMan), File S3: Forest plot of the overall pooled analysis, File S4: GRADE analyses, File S5: Dose response, File S6: Age groups, File S7: Trials with *H. pylori* eradication therapy, File S8: Low risk of bias, File S9: Intention-to-treat analyses, File S10: Search strategy.
