# Supplementary material for: Probiotics for the Prevention of Antibiotic-Associated Diarrhea in Outpatients—A Systematic Review and Meta-Analysis
Source: Antibiotics (Basel). 2017 Oct 12;6(4):21. doi: 10.3390/antibiotics6040021 (PMC5745464; doi:10.3390/antibiotics6040021)
Supplement: Supplementary file 1 [file antibiotics-06-00021-s001.zip › Supplementary Materials - antibiotics/S10_search strategy.docx]

Supplementary Materials

## Text box S10: PubMed Search Strategy

(((((((((("**Probiotics**"[Mesh] OR ("**probiotics**"[MeSH Terms] OR "**probiotics**"[All Fields])) OR ("**probiotics**"[MeSH Terms] OR "**probiotics**"[All Fields] OR "**probiotic**"[All Fields])) OR **bifidobacteria**[All Fields]) OR ("**bifidobacterium**"[MeSH Terms] OR "**bifidobacterium**"[All Fields])) OR ("**lactobacillaceae**"[MeSH Terms] OR "**lactobacillaceae**"[All Fields])) OR **lactobacilli**[All Fields]) OR ("**lactobacillus**"[MeSH Terms] OR "**lactobacillus**"[All Fields])) OR ("**saccharomyces**"[MeSH Terms] OR "**saccharomyces**"[All Fields])) AND (((((("**prevention and control**"[Subheading] OR ("**prevention and control**"[Subheading] OR ("**prevention**"[All Fields] AND "**control**"[All Fields]) OR "**prevention and control**"[All Fields] OR "**prevention**"[All Fields])) OR **preventing**[All Fields]) OR **preventive**[All Fields]) OR ("**prevention and control**"[Subheading] OR ("**prevention**"[All Fields] AND "**control**"[All Fields]) OR "**prevention and control**"[All Fields] OR "**prophylaxis**"[All Fields])) OR ("**prevention and control**"[Subheading] OR ("**prevention**"[All Fields] AND "**control**"[All Fields]) OR "**prevention and control**"[All Fields] OR "**control**"[All Fields] OR "**control groups**"[MeSH Terms] OR ("**control**"[All Fields] AND "**groups**"[All Fields]) OR "**control groups**"[All Fields])) OR **controlling**[All Fields])) AND (((("**Anti-Bacterial Agents**"[Mesh] OR ("**anti-bacterial agents**"[Pharmacological Action] OR "**anti-bacterial agents**"[MeSH Terms] OR ("**anti-bacterial**"[All Fields] AND "**agents**"[All Fields]) OR "**anti-bacterial agents**"[All Fields] OR ("**anti**"[All Fields] AND "**bacterial**"[All Fields] AND "**agents**"[All Fields]) OR "**anti bacterial agents**"[All Fields])) OR ("**anti-bacterial agents**"[Pharmacological Action] OR "**anti-bacterial agents**"[MeSH Terms] OR ("**anti-bacterial**"[All Fields] AND "**agents**"[All Fields]) OR "**anti-bacterial agents**"[All Fields] OR "**antibiotic**"[All Fields])) OR ("**anti-bacterial agents**"[Pharmacological Action] OR "**anti-bacterial agents**"[MeSH Terms] OR ("**anti-bacterial**"[All Fields] AND "**agents**"[All Fields]) OR "**anti-bacterial agents**"[All Fields] OR "**antibiotics**"[All Fields])) OR **antibiotherapy**[All Fields])) AND ((((((((("**Diarrhea**"[Mesh] OR ("**diarrhoea**"[All Fields] OR "**diarrhea**"[MeSH Terms] OR "**diarrhea**"[All Fields])) OR (**loose**[All Fields] AND ("**feces**"[MeSH Terms] OR "**feces**"[All Fields] OR "**stool**"[All Fields]))) OR ("**diarrhea**"[MeSH Terms] OR "**diarrhea**"[All Fields] OR ("**loose**"[All Fields] AND "**stools**"[All Fields]) OR "**loose stools**"[All Fields])) OR ("**diarrhea**"[MeSH Terms] OR "**diarrhea**"[All Fields] OR ("**watery**"[All Fields] AND "**stool**"[All Fields]) OR "**watery stool**"[All Fields])) OR ("**diarrhea**"[MeSH Terms] OR "**diarrhea**"[All Fields] OR ("**watery**"[All Fields] AND "**stools**"[All Fields]) OR "**watery stools**"[All Fields])) OR (**fluid**[All Fields] AND ("**feces**"[MeSH Terms] OR "**feces**"[All Fields] OR "**stool**"[All Fields]))) OR (**fluid**[All Fields] AND ("**feces**"[MeSH Terms] OR "**feces**"[All Fields] OR "**stools**"[All Fields]))) OR (("**running**"[MeSH Terms] OR "**running**"[All Fields]) AND ("**feces**"[MeSH Terms] OR "**feces**"[All Fields] OR "**stool**"[All Fields]))) OR (("**running**"[MeSH Terms] OR "**running**"[All Fields]) AND ("**feces**"[MeSH Terms] OR "**feces**"[All Fields] OR "**stools**"[All Fields])))
