# Supplementary material for: Probiotics for the Prevention of Antibiotic-Associated Diarrhea in Outpatients—A Systematic Review and Meta-Analysis
Source: Antibiotics (Basel). 2017 Oct 12;6(4):21. doi: 10.3390/antibiotics6040021 (PMC5745464; doi:10.3390/antibiotics6040021)
Supplement: Supplementary file 1 [file antibiotics-06-00021-s001.zip › Supplementary Materials - antibiotics/S1_study flow diagram.docx]

Supplementary Materials

## Figure S1: Study flow diagram

Records identified through MEDLINE/PubMed

(n=637)

Records screened

(n=637)

Records excluded (title and abstract irrelevant to the review title)

(n=584)

Full-text articles assessed for eligibility

(n=53)

Full-text articles excluded

(n=36)

for the following reasons:

Design (not a RCT)

(n=1)

Setting (studies including inpatients)

(n=31)

Outcome (not reporting on AAD)

(n=4)

Studies included in quantitative synthesis (meta-analysis)

(n=17)
