# Supplementary material for: Probiotics for the Prevention of Antibiotic-Associated Diarrhea in Outpatients—A Systematic Review and Meta-Analysis
Source: Antibiotics (Basel). 2017 Oct 12;6(4):21. doi: 10.3390/antibiotics6040021 (PMC5745464; doi:10.3390/antibiotics6040021)
Supplement: Supplementary file 1 [file antibiotics-06-00021-s001.zip › Supplementary Materials - antibiotics/S2_characteristics of included studies (RevMan).pdf]

Characteristics of studies

Characteristics of included studies

Tankanow et al., 1990

**Methods**	Double-blind RCT<br>ITT: no
**Participants**	N = 60 enrolled<br>Age group: Children 5mo-6y (mean age: 2.4y)<br>Diagnosis: Otitis media, pharyngitis<br>Setting: Outpatients (a local pediatric practice)<br>Country: USA
**Interventions**	Probiotic(s): *Lactobacillus acidophilus, Lactobacillus bulgaricus,* Dosage: 5.1 x 10^8 CFU, 4 times daily, Duration of treatment: 10 days<br>Control: Placebo<br>Antibiotic(s): Amoxicillin
**Outcomes**	Primary outcome: Incidence of diarrhea<br>Definition of diarrhea: 1 or more abnormally loose bowel movements/day throughout the study period of 1-10 days (parental reports).
**Notes**	Reference:<br>Tankanow RM, Ross MB, Ertel IJ, Dickinson DG, McCormick LS, Garfinkel JF. A double-blind, placebo-controlled study of the efficacy of Lactinex in the prophylaxis of amoxicillin-induced diarrhea. Dicp. 1990;24(4):382-4.

Risk of bias table

**Bias**	**Authors' judgement**	**Support for judgement**
Random sequence generation (selection bias)	Unclear risk	Randomization was provided by the manufacturer of the probiotic solution, however researchers did not explain further
Allocation concealment (selection bias)	Unclear risk	Not described
Blinding of participants and personnel (performance bias)	High risk	The study is "double-blinded", however researchers did not explain further. The randomization and blinding codes were provided by the manufacturer of the probiotic solution.
Blinding of outcome assessment (detection bias)	Unclear risk	Not decribed
Incomplete outcome data (attrition bias)	High risk	22/60 (36.7%) did not complete the study. 5 patients were lost to follow-up and 17 patients dropped out of the study within the first 5 days and their data were not evaluated. Reasons for dropping out of the study included children refusing to take the study medication due to taste, adverse reactions, noncompliance, and parents not comfortable with having children in a study.<br>The final number of subjects were 15 in the probiotic group and 23 in the placebo group.
Selective reporting (reporting bias)	Low risk	All expected outcomes were reported.
Other bias	High risk	This study was supported in full by Hynson, Westcott, and Dunning Products. They supplied the Lactinex granules, placebo and blinding and randomization codes.

Arvola et al., 1999

**Methods**	RCT<br>ITT: no
**Participants**	N = 167 enrolled<br>Age group: Children (age range not mentioned, but mean age: 4.5y)<br>Diagnosis: Acute respiratory infections<br>Setting: Outpatients (Health Care Centers - City of Tampere and Tampere University Hospital)<br>Country: Finland
**Interventions**	Probiotic(s): *Lactobacillus rhamnosus GG*, Dosage: 2 x 10^{10} CFU, twice daily, Duration of treatment: 7-10 days<br>Control: Placebo<br>Antibiotic(s):<br>Penicillin<br>Amoxicillin<br>Cephalosporins<br>Erythromycin

	Trimetoprim-sulpha
**Outcomes**	Primary outcome: Diarrhea during the first 2 weeks after the beginning of the antimicrobial treatment<br>Definition of diarrhea: at least 3 watery or loose stools/day for a minimum of 2 consecutive days<br>Secoundary outcome: Mean duration of diarrhea
**Notes**	Reference:<br>Arvola T, Laiho K, Torkkeli S, Mykkanen H, Salminen S, Maunula L, et al. Prophylactic Lactobacillus GG reduces<br>antibiotic-associated diarrhea in children with respiratory infections: a randomized study. Pediatrics. 1999;104(5):e64.

Risk of bias table

**Bias**	**Authors' judgement**	**Support for judgement**
Random sequence generation (selection bias)	Low risk	"Randomized by means of a computer program"
Allocation concealment (selection bias)	Unclear risk	Not described
Blinding of participants and personnel (performance bias)	Low risk	"Lactobacillus GG and placebo capsules were indistinguishable in appearance and taste when opened"
Blinding of outcome assessment (detection bias)	Unclear risk	Not described
Incomplete outcome data (attrition bias)	High risk	48/167 (28,7%) patients were lost to follow-up:<br>"Of the patients, 20 receiving placebo and 28 receiving Lactobacillus GG during antimicrobial treatment were lost to follow-up or discontinued because of difficulties in the transportation of the study samples. Therefore, the final study population consisted of 119 children."
Selective reporting (reporting bias)	Low risk	All expected outcomes were reported
Other bias	Low risk	"This study was supported financially by the Finnish Foundation for Gastroenterological Research, the Medical Research Fund of Tampere University Hospital, the Emil Aaltonen Foundation, and the Academy of Finland". The study appears to be free of other sources of bias.

Vanderhoof et al., 1999

**Methods**	Double-blind RCT<br>ITT: no
**Participants**	N = 202 enrolled<br>Age group: Children 6mo-10y (mean age: 4.0y)<br>Diagnosis: Otitis, pharyngitis, bronchitis, dermatologic, sinusitis and other<br>Setting: Outpatients (private primary care pediatric practice)<br>Country: USA
**Interventions**	Probiotic(s): *Lactobacillus rhamnosus GG*<br>Dosage:<br>Children <12kg:<br>1×10^{10} CFU, once daily<br>Children >12kg:<br>2×10^{10}, once daily<br>Duration of treatment: 10 days<br>Control: Placebo<br>Antibiotic(s):<br>Amoxicillin<br>Amoxicillin + clavulanate potassium<br>Cefprozil<br>Clarithomycin<br>and others
**Outcomes**	Primary outcome: Incidence of antibiotic-associated diarrhea<br>Definition of diarrhea: The presence of at least 2 liquid stools/day on at least 2 observation periods during the course of the study<br>Secoundary outcome: Mean duration of diarrhea
**Notes**	Reference:<br>Vanderhoof JA, Whitney DB, Antonson DL, Hanner TL, Lupo JV, Young RJ. Lactobacillus GG in the prevention of antibiotic-associated diarrhea in children. J Pediatr. 1999;135(5):564-8.

Risk of bias table

Bias	Authors' judgement	Support for judgement
Random sequence generation (selection bias)	Low risk	Computer-generated randomization table
Allocation concealment (selection bias)	Low risk	Randomization and blinding codes were kept by the supplier until all data were collected
Blinding of participants and personnel (performance bias)	Low risk	"The LGG and placebo were packed in identical bottles with identical capsule covers"
Blinding of outcome assessment (detection bias)	Low risk	Randomization and blinding codes were kept by the supplier until all data were collected
Incomplete outcome data (attrition bias)	Low risk	14/202 (6.9%) participants did not complete the study:<br>"14 failed to complete the study, primarily because of antibiotic noncompliance or inability of the investigators to contact the primary caregiver at the assigned follow-up time. None of the participants failed to complete the 10-day course of antibiotics because of a change in stool consistency or frequency. There were no failures resulting from untoward effects of either LGG or placebo. Both active and placebo groups were similar for age distribution, sex, and type of antibiotics, and all who completed the study had no difficulty consuming the prescribed amount"
Selective reporting (reporting bias)	Low risk	All expected outcomes were reported.
Other bias	High risk	The study was supported by a grant from CAG Nutrition, a division of ConAgra, Inc of Omaha, Nebraska. They also supplied the study product. Dr Vanderhoof serves as a consultant for ConAgra.

Erdeve et al., 2004

**Methods**	RCT in two parts (two different antibiotics)<br>ITT: no
**Participants**	N = 466 enrolled<br>Age group: Children (mean age not mentioned, but ages 1-15y were included)<br>Diagnosis: Not mentioned<br>Setting: Outpatients (otherwise not specified)<br>Country: Turkey
**Interventions**	Probiotic(s): *Saccharomyces boulardii*, Dosage: not mentioned, Duration of treatment: not mentioned<br>Control: Antibiotics-only<br>Antibiotic(s):<br>1) Sulbactam-ampicillin (N=234)<br>2) Azithromycin (N=232)
**Outcomes**	Primary outcome: Incidence of diarrhea<br>Definition of diarrhea: 3 or more watery stools/day during antibiotic treatment
**Notes**	Reference:<br>Erdeve O, Tiras U, Dallar Y. The probiotic effect of Saccharomyces boulardii in a pediatric age group. J Trop Pediatr.<br>2004;50(4):234-6.

Risk of bias table

Bias	Authors' judgement	Support for judgement
Random sequence generation (selection bias)	Unclear risk	Randomization not described
Allocation concealment (selection bias)	Unclear risk	Not described
Blinding of participants and personnel (performance bias)	Unclear risk	Not described
Blinding of outcome assessment (detection bias)	Unclear risk	Not described
Incomplete outcome data (attrition bias)	High risk	187/653 (28.6%) were lost to follow-up or withdrawn from the study, but researchers did not explain further
Selective reporting (reporting bias)	Low risk	All expected outcomes were reported
Other bias	Low risk	No mention of funding. The study seems to be free of other souces of bias.

Duman et al., 2005

**Methods**	Open-label RCT<br>ITT: no
**Participants**	N = 389 enrolled<br>Age group: Adults (age range not mentioned, but mean age: 45.68y)<br>Diagnosis: H. pylori infection<br>Setting: Outpatients (ambulatory outpatients from nine hospitals)<br>Country: Turkey
**Interventions**	Probiotic(s): *Saccharomyces boulardii*, Dosage: 500 mg, twice daily, Duration of treatment: 14 days<br>Control: Antibiotics-only<br>Antibiotic(s):<br>(H. pylori eradication)<br>Claritromycin<br>Amoxicillin
**Outcomes**	Primary outcome: Incidence of diarrhea during and following the antibiotic treatment<br>Definition of diarrhea: A change in bowel habits with at least 3 semi-solid or watery bowel movements/day for at least 2 consecutive days
**Notes**	Reference:<br>Duman DG, Bor S, Ozutemiz O, Sahin T, Oguz D, Istan F, et al. Efficacy and safety of Saccharomyces boulardii in prevention of antibiotic-associated diarrhoea due to Helicobacterpylori eradication. Eur J Gastroenterol Hepatol. 2005;17(12):1357-61.

Risk of bias table

Bias	Authors' judgement	Support for judgement
Random sequence generation (selection bias)	Unclear risk	Subjects "were randomly assigned", however researchers did not explain further
Allocation concealment (selection bias)	High risk	Open-label
Blinding of participants and personnel (performance bias)	High risk	Open-label
Blinding of outcome assessment (detection bias)	High risk	Open-label
Incomplete outcome data (attrition bias)	Unclear risk	Overall, 376 (96.7%) completed the study. 13 patients, 5 in the control group and 8 in the treatment group, did not complete the study. The number of drop-outs was not different between the groups. The reasons for not completing the study were as follows: 8 patients (3 in the control group, 5 in the treatment group) were not compliant with the study protocol, 2 patients (1 in each group) stopped taking their medications because of adverse events and 3 patients were lost to follow-up. The reported adverse events, which resulted in discontinuation of all the medications, were skin reaction in 1 patient in the treatment group and palpitation in the control group patient. Compliance with the study drug was complete in the rest of the patients of the treatment group. The data were missing in four patients during the treatment and the follow-up periods. Diarrhea duration could not be assessed in 6 patients during the treatment period due to missing records.
Selective reporting (reporting bias)	Low risk	All expected outcomes were reported
Other bias	Low risk	No mention of funding. The study seems to be free of other sources of bias.

Park et al., 2007

**Methods**	RCT<br>ITT: yes
**Participants**	N = 352 enrolled<br>Age group: Adults (age range not mentioned, but mean age: 45.2y)<br>Diagnosis: H. pylori infection<br>Setting: Outpatients (otherwise not specified)<br>Country: South Korea
**Interventions**	Probiotic(s): *Bacillus subtilis, Streptococcus faecium*<br>Dosage:<br>2 capsules three times a day:<br>2.5×10^9 CFU (Bacillus subtilis),<br>22.5×10^9 CFU (Streptococcus

	faecium)<br>Duration of treatment: 8 weeks<br>Control: Antibiotics-only<br>Antibiotic(s):<br>(H. pylori eradication)<br>Claritromycin<br>Amoxicillin
**Outcomes**	Primary outcome: Efficacy of probiotic supplementation in H. pylori eradication (eradication rates)<br>Secondary outcome(s): Incidence of side effects during antibiotic treatment (including diarrhea, nausea, and others).<br>Definition of diarrhea: Not specified (self-report).
**Notes**	Reference:<br>Park SK, Park DI, Choi JS, Kang MS, Park JH, Kim HJ, et al. The effect of probiotics on Helicobacter pylori eradication. Hepatogastroenterology. 2007;54(79):2032-6.

Risk of bias table

Bias	Authors' judgement	Support for judgement
Random sequence generation (selection bias)	Unclear risk	Participants were randomized, however researchers did not explain further
Allocation concealment (selection bias)	Low risk	"Randomization was performed using opaque envelopes containing study codes that had been prepared by a member of the research staff not directly involved in this study"
Blinding of participants and personnel (performance bias)	High risk	No blinding of participants or personnel
Blinding of outcome assessment (detection bias)	Unclear risk	Not described
Incomplete outcome data (attrition bias)	Low risk	16/352 (4.5%) did not complete the study: 13 patients were lost to follow-up (3 in the probiotic group and 10 in the control group), and 3 patients (1 in the probiotic group and 2 in the control group) newly diagnosed with malignancy were excluded for treatment.
Selective reporting (reporting bias)	Low risk	All expected outcomes were reported
Other bias	Low risk	No mention of funding. The study seems to be free from other sources of bias.

Conway et al., 2007

**Methods**	RCT with three arms<br>ITT: yes
**Participants**	N = 419 enrolled<br>Age group: Children and adults >1y (mean age: 37.8y)<br>Diagnosis: Not mentioned<br>Setting: Outpatients (a single primary care general practice surgery)<br>Country: England
**Interventions**	Probiotic(s): *Lactobacillus acidophilus, Streptococcus thermophilus, Bifidobacterium animalis lactis*, Dosage: 10^9 CFU, once daily, Duration of treatment: 12 days<br>Control: Antibiotics-only<br>Antibiotic(s):<br>Not mentioned
**Outcomes**	Primary outcome: Incidence of diarrhea<br>Definition of diarrhea: 3 or more loose stools/day over at least 2 consecutive days during the 12-day follow-up period
**Notes**	Reference:<br>Conway S, Hart A, Clark A, Harvey I. Does eating yogurt prevent antibiotic-associated diarrhoea? A placebo-controlled randomised controlled trial in general practice. Br J Gen Pract. 2007;57(545):953-9.

Risk of bias table

Bias	Authors' judgement	Support for judgement
Random sequence generation (selection bias)	Low risk	Random number table
Allocation concealment (selection bias)	Low risk	Sealed, opaque envelopes

Blinding of participants and personnel (performance bias)	High risk	Blinding for the two groups allocated to yogurt but no blinding of control group (antibiotics only)
Blinding of outcome assessment (detection bias)	Low risk	Assessor was blind to the patient's treatment allocation
Incomplete outcome data (attrition bias)	Low risk	"12 patients did not receive one of the two study yogurts due to administrative error. 38/407 (9%) were lost to follow-up. Of those 38, 12 had bio yoghurt, 9 had commercial yogurt and 17 had no yogurt. The total number of patients for whom there was complete follow-up was 369 (91% of 407)"
Selective reporting (reporting bias)	Low risk	All expected outcomes were reported
Other bias	Low risk	Yeo Valley Organics supplied the yogurt. An Enterprise Award from Eastern Region NHS Executive covered locum and other costs. The study seems to be free of other sources of bias.

Cindoruk et al., 2007

**Methods**	RCT<br>ITT: no
**Participants**	N = 124 enrolled<br>Age group: Adults (age range not mentioned, but mean age: 48.0y)<br>Diagnosis: H. pylori infection<br>Setting: Outpatients (otherwise not specified)<br>Country: Turkey
**Interventions**	Probiotic(s): *Saccharomyces boulardii*, Dosage: 500mg, twice daily, Duration of treatment: 14 days<br>Control: Placebo<br>Antibiotic(s):<br>(H. pylori eradication)<br>Claritromycin<br>Amoxicillin
**Outcomes**	Primary outcome: incidence of adverse effects related to *H. pylori* eradication (including diarrhea, nausea, epigastric discomfort, taste disturbance, urticaria and abdominal gas).<br>Definition of diarrhea: not specified (modified *De Boer* questionnaire categorizing diarrhea into "none", "mild", "moderate" and "severe").
**Notes**	Reference:<br>Cindoruk M, Erkan G, Karakan T, Dursun A, Unal S. Efficacy and safety of Saccharomyces boulardii in the 14-day triple anti-Helicobacter pylori therapy: a prospective randomized placebo-controlled double-blind study. Helicobacter. 2007;12(4):309-16.

Risk of bias table

**Bias**	**Authors' judgement**	**Support for judgement**
Random sequence generation (selection bias)	Low risk	"Randomization was done using computer-based random numbers"
Allocation concealment (selection bias)	Unclear risk	Not described
Blinding of participants and personnel (performance bias)	Low risk	"The manufacturer medical company gave placebo sachets. Placebo was administered in the same amount of sachets of probiotic schemes (250 mg, b.i.d.). Boxes containing active study treatments and placebo were identical in shape and color, and contained the same number of sachets. No trademark identifications were present, either on the probiotic or on the placebo sachets"
Blinding of outcome assessment (detection bias)	Low risk	"While the questionaire was performed, both the assessor and the subjects were blind to the treatment arm".
Incomplete outcome data (attrition bias)	Low risk	All participants completed the study
Selective reporting (reporting bias)	Low risk	All expected outcomes were reported
Other bias	Low risk	No mention of funding. The study seems to be free of other sources of bias.

Kim et al., 2008

**Methods**	Open-label RCT<br>ITT: yes
**Participants**	N = 347 enrolled<br>Age group: Adults 18-85y (mean age: 48.1y)<br>Diagnosis: H. pylori<br>Setting: Outpatients (Seoul National University Bundang Hospital)

	Country: South Korea
**Interventions**	Probiotic(s):<br>*Lactobacillus acidophilus, Lactobacillus casei, Bifidobacterium longum, Streptococcus thermophilus*<br>Dosage:<br>One bottle (150ml) per day:<br>>1 x 10^5 CFU/mL (L. acidophilus),<br>>1 x 10^5 CFU/mL (L. casei),<br>>1 x 10^6 CFU/mL (B. longum),<br>>1 x 108 CFU/mL (S. themophilus)<br>Duration of treatment: at least 3 weeks<br>Control: Antibiotics-only<br>Antibiotic(s):<br>(H. pylori eradication)<br>Claritromycin<br>Amoxicillin
**Outcomes**	Primary outcome: Eradication rates<br>Secondary outcomes: Adverse events (including diarrhea, metallic taste, epigastric pain, nausea and others).<br>Definition of diarrhea: Not specified other than categorized in groups ("none", "mild", "moderate", "severe").
**Notes**	Reference:<br>Kim MN, Kim N, Lee SH, Park YS, Hwang JH, Kim JW, et al. The effects of probiotics on PPI-triple therapy for Helicobacter pylori eradication. Helicobacter. 2008;13(4):261-8.

Risk of bias table

**Bias**	**Authors' judgement**	**Support for judgement**
Random sequence generation (selection bias)	High risk	Patients were randomized into two groups based on chart numbers, i.e. when 'odd' the patient was enrolled in the triple-plus-yogurt-group and when 'even' in triple-only group.
Allocation concealment (selection bias)	High risk	Open-label
Blinding of participants and personnel (performance bias)	High risk	Open-label
Blinding of outcome assessment (detection bias)	High risk	Open-label
Incomplete outcome data (attrition bias)	Low risk	A total of 25/347 (7.2%) were lost to follow-up (12 in the probiotic group and 13 in the treatment group). 2 subjects in the probiotic group and 1 in the control group were non-compliant. 2 subjects in the probiotic group and 1 in the control group discontinued therapy due to adverse events.
Selective reporting (reporting bias)	Low risk	All expected outcomes were reported
Other bias	Low risk	This study was supported by the Korean Health 21 R&D Project, Ministry of Health and Welfare, Republic of Korea. The study seems to be free of other sources of bias.

Imase et al., 2008

**Methods**	Open-label RCT<br>ITT: ?
**Participants**	N = 19 enrolled<br>Age group: Adults (age range 32-71y)<br>Diagnosis: H. pylori infection<br>Setting: Outpatients<br>Country: Japan
**Interventions**	Probiotic(s): *Clostridium butyricum*<br>Dosage:<br>1 x 10^7 CFU per tablet<br>Group B:<br>2 tablets, three times daily,<br>Group C:<br>4 tablets, three times daily<br>Duration of treatment: 7 days<br>Control: Antibiotics-only<br>Antibiotic(s):<br>(H. pylori eradication)<br>Claritromycin

	Amoxicillin
**Outcomes**	Primary outcome: eradication rates, incidence of diarrhea and soft stools<br>Definition of diarrhea: "loose or mostly loose stools", not specified further
**Notes**	Reference:<br>Imase K, Takahashi M, Tanaka A, Tokunaga K, Sugano H, Tanaka M, et al. Efficacy of Clostridium butyricum preparation concomitantly with Helicobacter pylori eradication therapy in relation to changes in the intestinal microbiota. Microbiol Immunol. 2008;52(3):156-61.

Risk of bias table

Bias	Authors' judgement	Support for judgement
Random sequence generation (selection bias)	Unclear risk	Randomization method not specified
Allocation concealment (selection bias)	Unclear risk	Not specified
Blinding of participants and personnel (performance bias)	High risk	Open-label
Blinding of outcome assessment (detection bias)	Unclear risk	Not mentioned
Incomplete outcome data (attrition bias)	Low risk	All patients completed the study
Selective reporting (reporting bias)	Low risk	
Other bias	Low risk	No mention of funding. The study seems to be free of other sources of bias.

Merenstein et al., 2009

**Methods**	Double-blind RCT<br>ITT: yes
**Participants**	N = 125 enrolled<br>Age group: Children 1-5y (mean age: 2.9y)<br>Diagnosis: Upper respiratory infections<br>Setting: Outpatients (primary care patients in the Washington, DC, metopolitan area)<br>Country: USA
**Interventions**	Probiotic(s): *Lactococcus lactis, Lactococcus plantarum, Lactococcus rhamnosus, Lactococcus casei, Lactococcus lactis subspecies diacetylactis, Leuconostoc cremoris, Bifidobacterium longum, Bifidobacterium breve, Lactobacillus acidophilus, Saccharomyces florentinus*<br>Dosage: One bottle (150ml) per day,<br>amount of CFU not mentioned<br>Duration of treatment: 10 days<br>Control: Placebo<br>Antibiotic(s):<br>Not mentioned
**Outcomes**	Primary outcome: Incidence of diarrhea during the 14-day follow-up period in children receiving antibiotics.<br>Definition of diarrhea: N/A (parental reports)
**Notes**	Reference:<br>Merenstein DJ, Foster J, D'Amico F. A randomized clinical trial measuring the influence of kefir on antibiotic-associated diarrhea: the measuring the influence of Kefir (MILK) Study. Arch Pediatr Adolesc Med. 2009;163(8):750-4.

Risk of bias table

Bias	Authors' judgement	Support for judgement
Random sequence generation (selection bias)	Low risk	"The randomization scheme was generated using permuted blocks with block size equal to 8"
Allocation concealment (selection bias)	Low risk	"It was impossible for research personnel involved with participants to adjust randomization or discern what drinks participants were receiving, ensuring true allocation concealment"
Blinding of participants and personnel (performance bias)	Low risk	"After production, both drinks were lime-flavored and had identical appearance, taste, and bottle size (150mL)"
Blinding of outcome assessment (detection bias)	Low risk	"All research personnel and staticians were blinded throughout the study, including during initial review of data"
Incomplete outcome data (attrition bias)	Low risk	"Loss to follow-up was exceptionally low. Only 4 participants in each group were unable to be contacted at the final follow-up on day 15"

Selective reporting (reporting bias)	Low risk	All expected outcomes were reported
Other bias	Low risk	This study was supported by Lifeway Foods, Inc, whose only role was to provide funding and product. No authors are associated with Lifeway Foods, Inc. The study seems to be free of other sources of bias.

De Vrese et al., 2011

**Methods**	Double-blind RCT with 3 parallel intervention groups<br>ITT: no
**Participants**	N = 88 enrolled<br>Age group: Adults 18-65y (mean age: 47.6y)<br>Diagnosis: H. pylori infection<br>Setting: Outpatients (otherwise not specified)<br>Country: Germany
**Interventions**	Probiotic(s): *Lactobacillus acidophilus LA-5, Bifidobacterium lactis BB-12*<br>Dosage:<br>>1 x 10^6 CFU/g,<br>125g, twice daily<br>Duration of treatment: 5 weeks<br>Control: Placebo<br>Antibiotic(s):<br>(H. pylori eradication)<br>Claritromycin<br>Amoxicillin
**Outcomes**	Gastrointestinal symptoms (including antibiotic-induced diarrhea) on day 1, 28, 35 and 56.<br>Definition of diarrhea: 3 or more watery stools for at least 1 day (where at least 1 episode lay within the eradication week).<br>Secoundary outcome: Mean duration of diarrhea
**Notes**	Reference:<br>de Vrese M, Kristen H, Rautenberg P, Laue C, Schrezenmeir J. Probiotic lactobacilli and bifidobacteria in a fermented milk product with added fruit preparation reduce antibiotic associated diarrhea and Helicobacter pylori activity. J Dairy Res. 2011;78(4):396-403.

Risk of bias table

**Bias**	**Authors' judgement**	**Support for judgement**
Random sequence generation (selection bias)	Unclear risk	"Random assignment to one of the 3 dietary groups", however researchers did not explain further
Allocation concealment (selection bias)	Unclear risk	Not described
Blinding of participants and personnel (performance bias)	Low risk	All three products (with and without probiotics) were mixed with the same fruit preparation in order to achieve identical appearance and taste. All test and control products were manufactured, prepacked and numbered randomly by the Dairy Education and Research Centre Oranienburg, Germany in order to fulfill the criteria of double-blinding.
Blinding of outcome assessment (detection bias)	Unclear risk	Not described
Incomplete outcome data (attrition bias)	Low risk	None of the 88 subjects who were finally included in the study dropped out, so there was no missing outcome data
Selective reporting (reporting bias)	Low risk	All expected outcomes were reported
Other bias	Low risk	The study was supported by Chr. Hansen GmbH, Nienburg, Germany J. Bauer GmbH & Co. KG, Wasserburg/Inn, Germany, Privat-Molkerei Borgmann GmbH & Co. KG, Coesfeld, Germany, NÖM AG, Molkerei, Baden bei Wien, Austria, Molkerei H. Strothmann GmbH, Gütersloh, Germany. The study seems to be free of other sources of bias.

Ojetti et al., 2012

**Methods**	RCT<br>ITT: yes
**Participants**	N = 90 enrolled<br>Age group: Adults 18-65y (mean age: 41.5y)<br>Diagnosis: H. pylori infection<br>Setting: Outpatients (trial performed at the Gastroenterology and Internal Medicine Departments of Gemelli

	Hospita of Rome)<br>Country: Italy
**Interventions**	Probiotic(s): *Lactobacillus reuteri*, Dosage: 1 x 10^8 CFU, three times daily, Duration of treatment: 14 days<br>Control: Antibiotics-only<br>Antibiotic(s):<br>(H. pylori eradication)<br>Levofloxacin<br>Amoxicillin
**Outcomes**	Primary outcome: Efficacy of L reuteri supplementation in H. pylori eradication<br>Secondary outcome(s): Incidence of GI-associated side effects during a second-line levofloxacin triple therapy (including diarrhea, nausea, and others).<br>Definition of diarrhea: Not specified other than categorized in groups ("none", "mild", "moderate", "severe").
**Notes**	Reference:<br>Ojetti V, Bruno G, Ainora ME, Gigante G, Rizzo G, Roccarina D, et al. Impact of Lactobacillus reuteri Supplementation on Anti-Helicobacter pylori Levofloxacin-Based Second-Line Therapy. Gastroenterol Res Pract. 2012;2012:740381.

Risk of bias table

Bias	Authors' judgement	Support for judgement
Random sequence generation (selection bias)	Low risk	"Permuted block randomization (1:1)"
Allocation concealment (selection bias)	Unclear risk	Not described
Blinding of participants and personnel (performance bias)	High risk	No blinding of participants or personnel
Blinding of outcome assessment (detection bias)	Unclear risk	Not described
Incomplete outcome data (attrition bias)	Low risk	All patients completed the study
Selective reporting (reporting bias)	Low risk	All expected outcomes were reported
Other bias	Low risk	The Catholic University Research Group on Gut Microflora is funded by The Fondazione Ricerca in Medicina. The study seems to be free from other sources of bias

Chatterjee et al., 2013

**Methods**	RCT<br>ITT: yes - "modified intention-to-treat analysis" (i.e. all subjects who had attended at least one post-baseline visit were considered evaluable)
**Participants**	N = 396 enrolled<br>Age group: Adults and elderly 18-70y (mean age not mentioned)<br>Diagnosis: URTI, LRTI, others<br>Setting: Outpatients (An outpatient setting in four tertiary care hospitals)<br>Country: India
**Interventions**	Probiotic(s): *Lactobacillus acidophilus La-5, Bifidobacterium Bb-12*<br>Dosage: 4 x 10^9 CFU<br>Duration of treatment: 14 days<br>Control: Placebo<br>Antibiotic(s):<br>Cefadroxil<br>Amoxycillin
**Outcomes**	Primary outcome: Incidence of AAD<br>Definition of diarrhea: Passage of at least 3 or more watery or loose stools/day for at least 2 consecutive days<br>Secoundary outcome: Mean duration of diarrhea
**Notes**	Reference:<br>Chatterjee S, Kar P, Das T, Ray S, Gangulyt S, Rajendiran C, et al. Randomised placebo-controlled double blind multicentric trial on efficacy and safety of Lactobacillus acidophilus LA-5 and Bifidobacterium BB-12 for prevention of antibioticassociated diarrhoea. J Assoc Physicians India. 2013;61(10):708-12.

Risk of bias table

Bias	Authors' judgement	Support for judgement
Random sequence generation (selection bias)	Low risk	"Randomisation was done using computer generated random number sequence with equal allocation ratio"
Allocation concealment (selection bias)	Low risk	"Allocation concealment was done by using sequentially numbered opaque sealed envelopes"
Blinding of participants and personnel (performance bias)	Low risk	"(...)The control group received identical looking placebo capsules"
Blinding of outcome assessment (detection bias)	Unclear risk	Not described
Incomplete outcome data (attrition bias)	Low risk	53/396 (13.4%) were lost to follow-up/withdrawn:<br>"Out of 22 non-evaluable subjects in probiotic arm - 18 were lost to follow-up and 4 were protocol deviators while out of 31 non-evaluable subjects in placebo arm 22 were lost to follow-up and rest (=9) were protocol deviators."
Selective reporting (reporting bias)	Low risk	All expected outcomes were reported
Other bias	Low risk	Study sponsored by Zydus Cadila Healthcare Ltd. India and Chr. Hansen A/S, Netherlands. The study seems to be free of other sources of bias.

Zojaji et al., 2013

**Methods**	RCT<br>ITT: no
**Participants**	N = 160 enrolled<br>Age group: Adults >15y (mean age: 47.1)<br>Diagnosis: H. pylori infection<br>Setting: Outpatients (A gastroenterology ward in Taleghani Hospital, a tertiary academic center in Tehran)<br>Country: Iran
**Interventions**	Probiotic(s): *Saccharomyces boulardiito*<br>Dosage: 250mg twice daily,<br>amount of CFU not mentioned<br>Duration of treatment: 14 days<br>Control: Antibiotics-only<br>Antibiotic(s):<br>(H. pylori eradication)<br>Claritromycin<br>Amoxicillin
**Outcomes**	Primary outcome: Efficacy of probiotic supplementation in H. pylori eradication (eradication rates)<br>Secondary outcome(s): Incidence of side effects during antibiotic treatment (including diarrhea, nausea, and others).<br>Definition of diarrhea: Not specified (self-report).
**Notes**	Reference:Zojaji H, Ghobakhlou M, Rajabalinia H, Ataei E, Jahani Sherafat S, Moghimi-Dehkordi B, et al. The efficacy and safety of adding the probiotic Saccharomyces boulardiito standard triple therapy for eradication of H.pylori: a randomized controlled trial. Gastroenterol Hepatol Bed Bench. 2013;6(Suppl 1):S99-s104.

Risk of bias table

Bias	Authors' judgement	Support for judgement
Random sequence generation (selection bias)	Unclear risk	Patients "were randomized into two treatment regimens", however researchers did not explain further
Allocation concealment (selection bias)	Unclear risk	Not described
Blinding of participants and personnel (performance bias)	High risk	No blinding of participants or personnel
Blinding of outcome assessment (detection bias)	Unclear risk	Not described
Incomplete outcome data (attrition bias)	Low risk	All 160 subjects were evaluated
Selective reporting (reporting bias)	Low risk	All expected outcomes were reported
Other bias	Low risk	This project is supported by Research Center for Gastroenterology and Liver Diseases. The study seems to be free of other sources of bias.

Fox et al., 2014

**Methods**	Double-blind RCT<br>ITT: no
**Participants**	N = 72 enrolled<br>Age group: Children 1-12y (mean age: 6.3y)<br>Diagnosis: ear infection, throat infection, chest infection, other/unknown<br>Setting: Outpatients (general practices and pharmacies in Launceston, Tasmania)<br>Country: Australia
**Interventions**	Probiotic(s): *Lactobacillus rhamnosus GG, Lactobacillus acidophilus LA-5, Bifidobacterium Bb-12*<br>Dosage:<br>5.2×10^9 CFU (L. rhamnosus)<br>5.9×10^9 CFU (B. Bb-12)<br>8.3×10^9 CFU (L. acidophilus LA-5)<br>Duration of treatment:<br>No. of days not mentioned (“From the start to the end of their antibiotic treatment”)<br>Control: Placebo<br>Antibiotic(s):<br>Beta-lactams<br>Macrolides<br>Tetracyclines
**Outcomes**	Primary outcome: Stool frequency and consistency, classified at different levels of diarrhea severity<br>Definition of diarrhea:<br>"A" (stool consistency >=5, >=2 stools/day for >=2 days)<br>"B" (stool consistency >=5, >=3 stools/day for >=2 days)<br>"C" (stool consistency >=6, >=2 stools/day for >=2 days)<br>"D" (stool consistency >=6, >=3 stools/day for >=2 days)
**Notes**	Reference:<br>Fox MJ, Ahuja KD, Robertson IK, Ball MJ, Eri RD. Can probiotic yogurt prevent diarrhoea in children on antibiotics? A double-blind, randomised, placebo-controlled study. BMJ Open. 2015;5(1):e006474.

Risk of bias table

**Bias**	**Authors' judgement**	**Support for judgement**
Random sequence generation (selection bias)	Low risk	"A statician generated independent allocation sequences and randomisation lists for each study site, using the random number generator in Microsoft Excel"
Allocation concealment (selection bias)	Low risk	"To ensure allocation concealment, an independent person oversaw the packaging and labelling of trial treatments based on the randomisation schedule"
Blinding of participants and personnel (performance bias)	Low risk	"All investigators, participants, outcome assessors and data analysts were blinded to the assigned treatment throughout the study" and "the yogurt was in 100g containers with identical labels. The yogurts were similar in taste but one yogurt was thinner in texture. Participants were only shown the yogurt they were going to use and did not have the opportunity to make a comparison"
Blinding of outcome assessment (detection bias)	Low risk	Outcome assessors were blinded
Incomplete outcome data (attrition bias)	Low risk	2/72 (2.8%) were lost to follow-up. Researchers did not mention which of the two groups they were from.
Selective reporting (reporting bias)	Low risk	All expected outcomes were reported
Other bias	Low risk	The study was supported by Parmalat Australia. They had no role in the formulation or conduct of the study or in the data analysis or interpretation. The study seems to be free of other souces of bias.

Olek et al., 2017

**Methods**	Double-blind RCT<br>ITT: no
**Participants**	N = 447 enrolled<br>Age group: Children 1-11y (mean age: 5.2y)<br>Diagnosis: respiratory tract infections, throat infections, ear infections, urinary tract infections<br>Setting: Outpatients (primary healthcare centers)<br>Country: Poland

**Interventions**	Probiotic(s): *Lactobacillus plantarum* DSM9843 (LP299V)<br>Dosage: 1 x 10^{10} CFU/capsule<br>Duration of treatment:<br>5-10 days during antibiotic treatment + 1 week after (±2 days)<br>Control: Placebo<br>Antibiotic(s):<br>Penicillins<br>Cephalosporins<br>Sulfometoksazole<br>Trimethoprim<br>Macrolides
**Outcomes**	Primary outcome: incidence of loose/watery stools<br>Secondary outcomes: incidence of AAD, mean number of loose/watery stools, incidence of abdominal symptoms, incidence of adverse events<br>Definition of diarrhea: >=3 loose/watery stools/24 hours starting after the initiation of antibiotic treatment
**Notes**	Reference:<br>Olek A, Woynarowski M, Ahren IL, Kierkus J, Socha P, Larsson N, et al. Efficacy and Safety of Lactobacillus plantarum DSM 9843 (LP299V) in the Prevention of Antibiotic-Associated Gastrointestinal Symptoms in Children-Randomized, Double-Blind, Placebo-Controlled Study. J Pediatr. 2017;186:82-6.

Risk of bias table

Bias	Authors' judgement	Support for judgement
Random sequence generation (selection bias)	Low risk	"The children were randomly allocated to LP299V or placebo following a 1:1 randomization in blocks of 4. The randomization list used for the labenling and allocation of the study product was generated using SAS Proc v 9.1"
Allocation concealment (selection bias)	Low risk	"The information about the allocation to specific study arm remained blind to patients, parents, and all members of the study team including the investigators, monitors, and data managers who assesed the study outcomes until all data were collected and verified"
Blinding of participants and personnel (performance bias)	Low risk	"The information about the allocation to specific study arm remained blind to patients, parents, and all members of the study team including the investigators, monitors, and data managers who assesed the study outcomes until all data were collected and verified" and "the placebo capsules had the same appearance, texture and taste as those with the active product but contained only potato starch"
Blinding of outcome assessment (detection bias)	Low risk	"The information about the allocation to specific study arm remained blind to patients, parents, and all members of the study team including the investigators, monitors, and data managers who assesed the study outcomes until all data were collected and verified"
Incomplete outcome data (attrition bias)	Low risk	"The following protocol violations caused withdrawal from per protocol analysis: withdrawal of the informed consent for continuation of therapy (18), noncompliance with the duration of the seperate study phases (11), noncompliance (<80% or >120% of recommended amount) with study product administration (10), intake of other probiotics, violation of inclusion/exclusion criteria (6)".
Selective reporting (reporting bias)	Low risk	All expected outcomes were reported
Other bias	Low risk	No mention of funding. The study seems to be free from other sources of bias.

Footnotes
