# Supplementary material for: Probiotics for the Prevention of Antibiotic-Associated Diarrhea in Outpatients—A Systematic Review and Meta-Analysis
Source: Antibiotics (Basel). 2017 Oct 12;6(4):21. doi: 10.3390/antibiotics6040021 (PMC5745464; doi:10.3390/antibiotics6040021)
Supplement: Supplementary file 1 [file antibiotics-06-00021-s001.zip › Supplementary Materials - antibiotics/S4_GRADE analysis.docx]

Supplementary Materials

## Table S4: GRADE Analysis

| **Probiotics for the prevention of antibiotic-associated diarrhea in outpatients**  **Bibliography:** | | | | | | | | | | | |
| --- | --- | --- | --- | --- | --- | --- | --- | --- | --- | --- | --- |
| **Certainty assessment** | | | | | | | **Summary of findings** | | | | |
| **№ of participants (studies) Follow-up** | **Risk of bias** | **Inconsistency** | **Indirectness** | **Imprecision** | **Publication bias** | **Overall certainty of evidence** | **Study event rates (%)** | | **Relative effect (95% CI)** | **Anticipated absolute effects** | |
|  |  |  |  |  |  |  | **With control** | **With probiotics** |  | **Risk with control** | **Risk difference with probiotics** |
| **Main outcome: Incidence of AAD** | | | | | | | | | | | |
| 3631 (17 RCTs) | serious ^a^ | serious ^b^ | not serious | not serious | strong association | ⨁⨁⨁◯ MODERATE | 319/1799 (17.7%) | 147/1832 (8.0%) | **RR 0.49** (0.36 to 0.67) | 177 per 1.000 | **90 fewer per 1.000** (113 fewer to 59 fewer) |
| **Main outcome: Incidence of AAD in strain-specific subgroup analyses** | | | | | | | | | | | |
| 1901 (8 RCTs) | serious ^c^ | not serious | not serious | not serious | strong association | ⨁⨁⨁⨁ HIGH | 176/929 (18.9%) | 81/972 (8.3%) | **RR 0.45** (0.34 to 0.60) | 189 per 1.000 | **104 fewer per 1.000** (125 fewer to 76 fewer) |
| **Secondary outcome: Incidence of AAD using the criteria defined by WHO** | | | | | | | | | | | |
| 1724 (7 RCTs) | serious ^a^ | not serious | not serious | not serious | strong association | ⨁⨁⨁⨁ HIGH | 108/848 (12.7%) | 55/876 (6.3%) | **RR 0.54** (0.36 to 0.82) | 127 per 1.000 | **59 fewer per 1.000** (82 fewer to 23 fewer) |
| **Secondary outcome: Incidence of adverse events** | | | | | | | | | | | |
| 2363 (10 RCTs) | serious ^a^ | serious ^d^ | serious ^e^ | not serious | strong association | ⨁⨁◯◯ LOW | 122/1180 (10.3%) | 122/1183 (10.3%) | **RD 0.00** (-0.02 to 0.02) | 103 per 1.000 | **103 fewer per 1.000** (105 fewer to 101 fewer) |

**CI:** Confidence interval; **RR:** Risk ratio

#### Explanations

a. Only three trials were assessed as having a ”ow risk of bias”. The risk of bias in most trials was rated as ”unclear”.

b. I^2^ is 58% with a *p* value of 0.001, suggesting moderate heterogeneity. While heterogeneity was explored, it could not be explained completely with *a priori* subgroup analyses.

c. The risk of bias in most trials was rated as ”unclear” or ”high”.

d. I^2^ is 66% with a *p* value of 0.002, suggesting substantial heterogeneity.

e. Due to widely varying definitions of adverse events, there is considerable indirectness in terms of outcomes.

Made with GRADEpro GDT: GRADEpro Guideline Development Tool [Software]. McMaster University, 2015 (developed by Evidence Prime, Inc.). Available from: <https://gradepro.org./>
