# Supplementary figures and images for: Probiotics for the Prevention of Antibiotic-Associated Diarrhea in Outpatients—A Systematic Review and Meta-Analysis
Source: Antibiotics (Basel). 2017 Oct 12;6(4):21. doi: 10.3390/antibiotics6040021 (PMC5745464; doi:10.3390/antibiotics6040021)

Supplementary Materials

## Figure S3: Overall pooled analysis of 17 RCTs


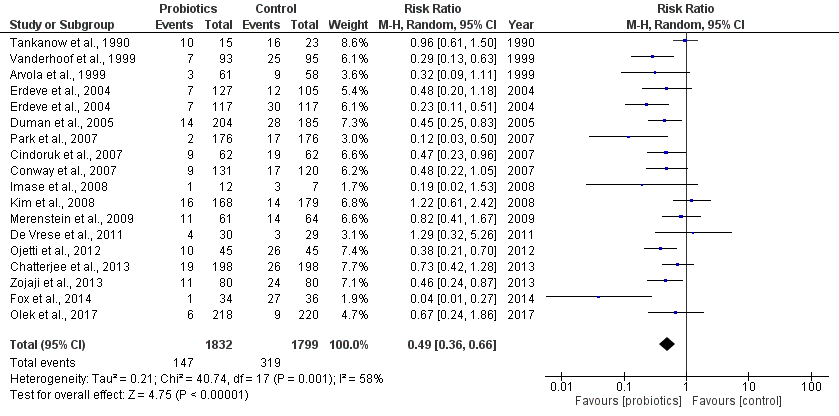

Supplement: Supplementary file 1 [file antibiotics-06-00021-s001.zip › Supplementary Materials - antibiotics/S3_Overall pooled analysis.docx]

Supplementary Materials

## Figure S5: High dose versus Low dose


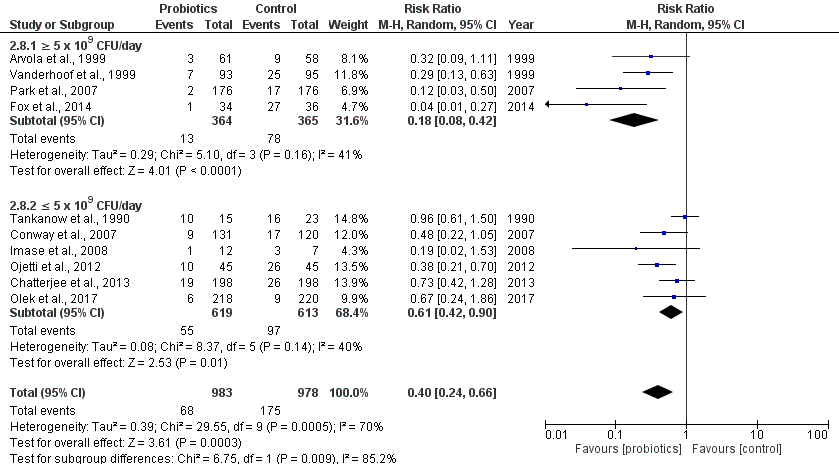

Supplement: Supplementary file 1 [file antibiotics-06-00021-s001.zip › Supplementary Materials - antibiotics/S5_dose response.docx]

Supplementary Materials

## Figure S6: Age groups


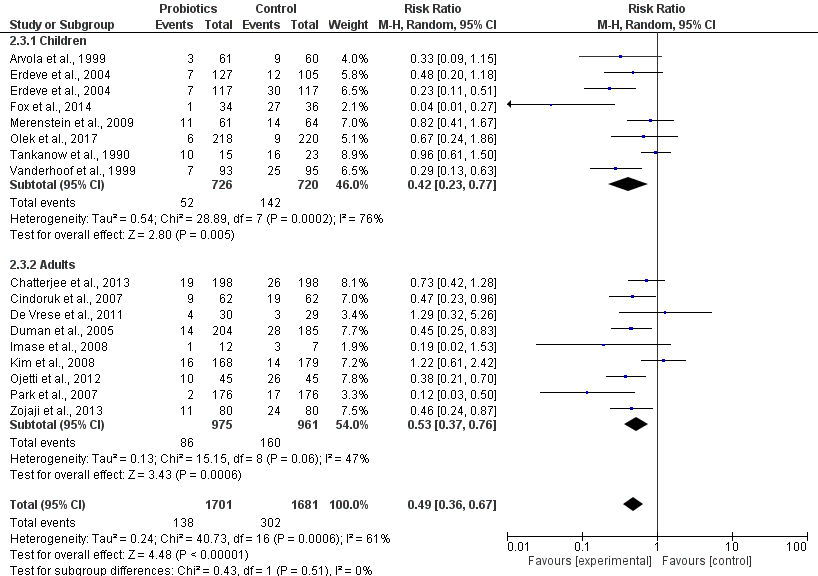

Supplement: Supplementary file 1 [file antibiotics-06-00021-s001.zip › Supplementary Materials - antibiotics/S6_age groups.docx]

Supplementary Materials

## Figure S7: Trials with *H. pylori eradication* therapy


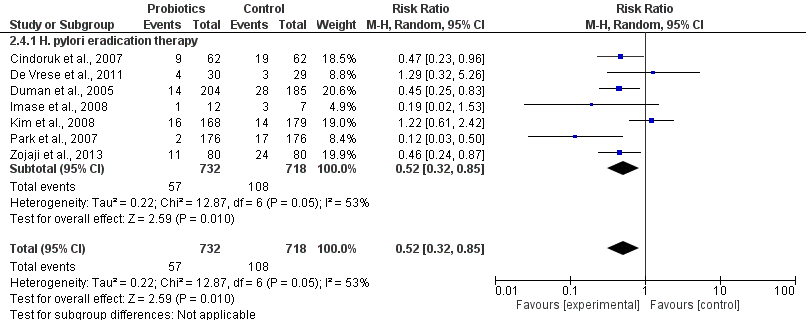

Supplement: Supplementary file 1 [file antibiotics-06-00021-s001.zip › Supplementary Materials - antibiotics/S7_Trials with H. pylori eradication therapy.docx]

Supplementary Materials

## Figure S8: Low risk of bias


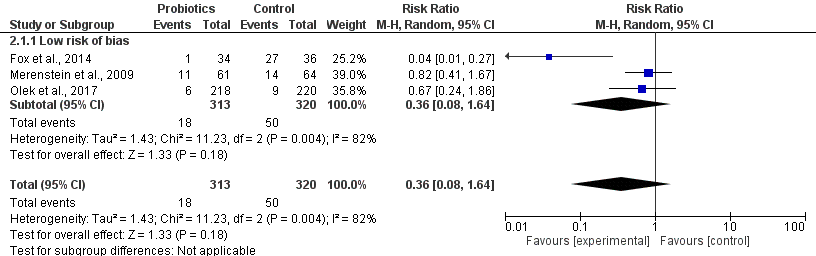

Supplement: Supplementary file 1 [file antibiotics-06-00021-s001.zip › Supplementary Materials - antibiotics/S8_Low risk of bias.docx]

Supplementary Materials

## Figure S9: Intention-to-treat analyses


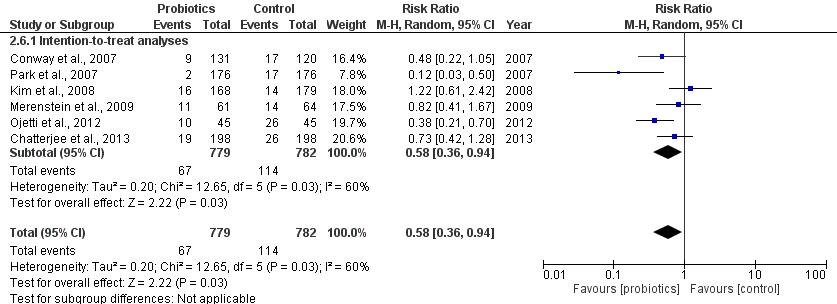

Supplement: Supplementary file 1 [file antibiotics-06-00021-s001.zip › Supplementary Materials - antibiotics/S9_Intention-to-treat analyses.docx]
